# MicroRNA and Diabetic Bone Disease

**DOI:** 10.1007/s11914-022-00731-0

**Published:** 2022-06-08

**Authors:** Souad Daamouch, Lejla Emini, Martina Rauner, Lorenz C. Hofbauer

**Affiliations:** Department of Medicine III and Center for Healthy Aging, Technische Universität Dresden, Fetscherstraße 74, 01307 Dresden, Germany

**Keywords:** MicroRNA, Type 1 diabetes mellitus, Type 2 diabetes mellitus, Bone fragility, Diabetic bone disease, Biomarkers

## Abstract

**Purpose of Review:**

The incidence of diabetes is increasing worldwide. Diabetes mellitus is characterized by hyperglycemia, which in the long-term damages the function of many organs including the eyes, the vasculature, the nervous system, and the kidneys, thereby imposing an important cause of morbidity for affected individuals. More recently, increased bone fragility was also noted in patients with diabetes. While patients with type 1 diabetes mellitus (T1DM) have low bone mass and a 6-fold risk for hip fractures, patients with type 2 diabetes mellitus (T2DM) have an increased bone mass, yet still display a 2-fold elevated risk for hip fractures. Although the underlying mechanisms are just beginning to be unraveled, it is clear that diagnostic tools are lacking to identify patients at risk for fracture, especially in the case of T2DM, in which classical tools to diagnose osteoporosis such as dual X-ray absorptiometry have limitations. Thus, new biomarkers are urgently needed to help identify patients with diabetes who are at risk to fracture.

**Recent Findings:**

Previously, microRNAs have received great attention not only for being involved in the pathogenesis of various chronic diseases, including osteoporosis, but also for their value as biomarkers.

**Summary:**

Here, we summarize the current knowledge on microRNAs and their role in diabetic bone disease and highlight recent studies on miRNAs as biomarkers to predict bone fragility in T1DM and T2DM. Finally, we discuss future directions and challenges for their use as prognostic markers.

## Introduction

According to the World Health Organization (WHO), more than 422 million people are suffering from diabetes mellitus worldwide and this number continues to increase each year [1]. The WHO estimates that 592 million adults worldwide will be affected by diabetes by 2035. Diabetes mellitus is a serious chronic disease characterized by high sugar levels in the blood that result from dysregulated insulin production, insulin utility, and its signaling pathway [2]. The two most common forms of diabetes mellitus are type 1 diabetes mellitus (T1DM) and type 2 diabetes mellitus (T2DM). While T1DM is an autoimmune disease that leads to destruction of the insulin-producing pancreatic beta cells and frequently occurs during childhood or young adulthood, T2DM accounts for up to 90% of diabetes and this number is expected to increase over time [3]. T2DM is characterized by insulin resistance and a relative lack of insulin production and action, which are, among others, facilitated by poor diet and lack of exercise [4]. Diabetes is linked to a high rate of morbidity and mortality. Considering that early stages of diabetes development are silent and asymptomatic, it is estimated that approximately 50% of the population are living with diabetes without being diagnosed [5]. Undiagnosed and delayed diagnosis frequently result in complications such as cardiovascular disease; neurological, renal, and ocular manifestations; as well as bone fragility, the latter which has only been recognized recently.

Due to the unique pathophysiology of T1DM and T2DM, also their manifestation in bone is distinct. While both are characterized by a higher fracture rate (6-fold in T1DM and 2.4-fold in T2DM), patients with T1DM have low bone mineral density (BMD), while patients with T2DM have normal or higher BMD [6, 7, 8•]. Thus, it becomes obvious that, in particular in patients with T2DM, BMD measurements are not useful to identify patients at risk for fracture. This calls for additional measures that help predict bone fragility in diabetics. Recently, microRNAs (miRNAs) have received much attention as biomarkers for various diseases including diabetes and osteoporosis [9]. miRNAs are small non-protein-coding RNA molecules that influence gene expression in addition to protein-coding genes and are readily found in all body fluids. Based on an increasing number of reports on miRNAs associated with T1DM and T2DM and bone fragility and their potential to help identify patients with diabetes with bone fragility, we here summarize the recent findings regarding their role in the pathophysiology of diabetic bone disease and their importance as biomarkers for an early diagnosis of diabetes and its bone-associated complications.

## miRNA

In 1993 Victor Ambros and Gary Ruvkun discovered small regulatory molecules in the nematode *Caenorhabditis elegans* today known as miRNA [10]. miRNA are short single-stranded non-coding RNA molecules consisting of 18–22 nucleotides. These non-coding RNAs function by regulating gene expression at post-transcriptional level by binding to the three prime untranslated regions of target mRNAs [11]. Many mRNAs can be targeted by one single miRNA influencing the expression of several genes involved in biological pathways. More than 2000 miRNAs have been reported in humans and are ubiquitously expressed in most cell types and tissues [12].

miRNAs are mainly detected in cells although a small number of miRNA have been detected in the extracellular environment and these miRNAs are known as circulating miRNA. Circulating miRNAs are present in various body fluids including saliva, tears, urine, cerebrospinal fluid, and follicular fluid. Additionally, miRNAs have been isolated from blood (serum and plasma) [13, 14]. The transportation, stability, and protection of circulating miRNA in bio-fluids are mediated through exosomes and microvesicles that are released from a variety of cell types [15].

miRNAs play a crucial role in biological processes such as cell growth, differentiation, and organ development. Irregularities of miRNA expression profiles caused either by genetic or environmental factors have been associated with human diseases, thus, proposing their potential as novel therapeutic targets. In the field of skeletal biology, studies have shown that osteoblast and osteoclast differentiation and function are regulated by miRNA highlighting their regulatory role in bone formation and bone resorption, bone remodeling, and bone repair [16]. miRNAs have also been shown to control tissues relevant for the development of diabetes including the pancreas, skeletal muscle, and adipose tissue. Abnormal miRNA expression levels have been found in sera and tissues from patients with T1DM, T2DM, and osteoporosis indicating their involvement in bone and metabolic disorders [15, 17]. Many of these dysregulated miRNAs are involved in pancreatic beta cell destruction [18–22], autoimmunity mechanisms [23–28], angiogenesis [23, 25], osteoporosis [14, 17, 29, 30], insulin resistance [31–33], vascular dysfunction [34–36], and hyperglycemia [20].

In the following chapters, we will present recent knowledge on the role of miRNA in the development of T1DM and T2DM bone disease, and on their utility as biomarkers to predict disease development, therapy response, and fracture risk.

## The Role of miRNA in T1DM and Bone Pathology

According to the American Diabetes Association, T1DM affects almost 1.6 million people in the USA. T1DM is an autoimmune disease in which the host immune system attacks pancreatic beta cells. These beta cells have a key role in the regulation of blood glucose as they produce insulin that allows blood glucose to enter into cells and to be used as a source of energy. Thus, T1DM is eventually characterized by absolute insulin deficiency as pancreatic beta cells are gradually destroyed that leads to hyperglycemia. Patients with T1DM present no clinical signs until massive destruction of 80 to 90% of beta cells occurs [5]. Typically, T1DM starts in early childhood [37] and as a chronic disease is maintained during lifetime. Patients frequently present symptoms including polydipsia, polyuria, a loss in body weight, and abnormal fatigue. Currently, the mainstay treatment is to substitute insulin. In addition, a healthy diet and physical exercise are key for good control of blood glucose levels. Besides these classical symptoms of T1DM, prolonged and poorly managed disease can lead to many complications, such as heart attack and strokes, retinopathy leading to blindness, and kidney failure. More recently, also osteoporosis and a 6-fold increased risk to hip fracture were observed in patients with T1DM [38•]. Mechanistically, the deficiency of the bone-anabolic hormone insulin is thought to be one of the main mechanisms leading to poor bone quality. However, the pathogenesis of T1DM skeletal complications is complex and multifactorial, and it has yet to be fully understood. Nonetheless, novel biomarkers could allow early diagnosis to prevent osteoporosis and its pathological signs.

**Fig. 1:**
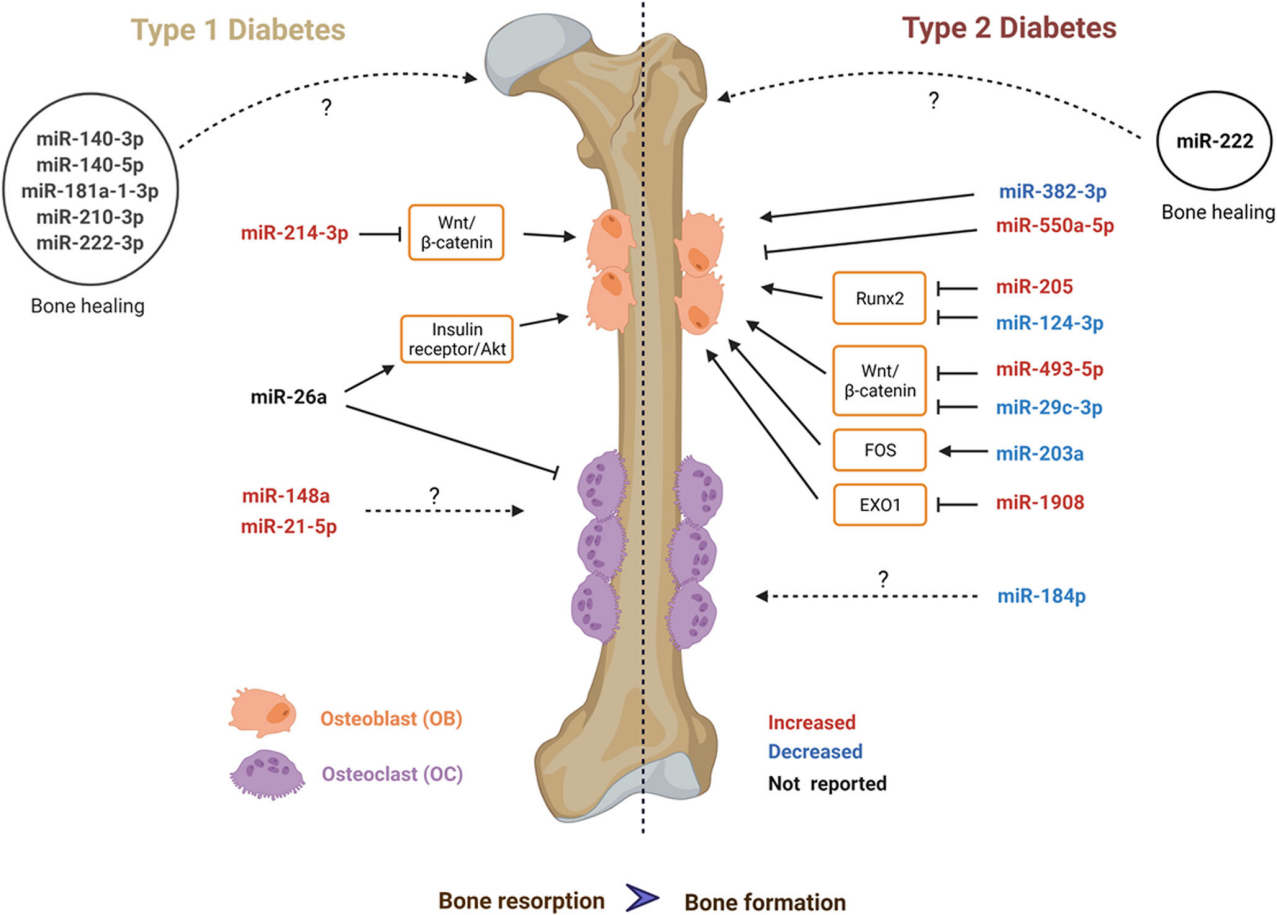
miRNAs involved in T1DM and T2DM bone disease. miRNAs involved in type 1 (left side) and type 2 (right side) diabetic bone disease. miRNAs that are upregulated in diabetic bone disease in humans or rodent models are indicated in red, downregulated ones in blue. Experimentally verified target genes are indicated in the boxes. For some regulated miRNAs, their role in diabetic bone disease is not yet known (indicated with dashed lines). miRNAs in circles have been identified during bone healing in diabetic rodent models with so far unknown underlying mechanisms. Created with BioRender.com

So far, only few studies have investigated the role of miRNAs in T1DM bone disease. One study investigated the expression of 6 pre-defined bone-related miRNAs in patients with T1DM [39••]. They analyzed the expression level of miR-21, miR-24, miR-27a, miR-148a, miR-214, and miR-375 in serum from 15 patients with T1DM versus 14 non-diabetic participants. Among these miRNAs, miR-148a-3p and miR-21-5p were increased in the blood of patients with T1DM compared to the non-diabetic group. Correlation analyses of those two miRNAs revealed no correlation with metabolic parameters such as the BMI or HbA1c; however, they were negatively correlated with BMD. Thus, these findings suggest their potential role as biomarkers for T1DM bone fragility (Fig. 1).

Even though miR-214 only tended to be increased in the serum of T1DM in the former study, another study validated its importance in the development of diabetic bone disease in mice and humans. Treatment of bone marrow mesenchymal cells with high glucose induced miR-214-3p expression and inhibited osteogenic differentiation via suppressing the Wnt/β-catenin pathway. Further, miR-214-3p knockout mice were protected from diabetic bone loss*.* Finally, the upregulation of miR-214-3p and downregulation of β-catenin was verified in bone specimens from patients with T1DM and osteoporosis compared to healthy subjects [40] (Fig. 1), highlighting the importance of miR-214-3p in T1DM bone disease.

Recently, the impact of miR-26a was investigated in a mouse model of T1DM bone disease, as this miRNA is specifically involved in osteogenic differentiation. Administration of miR-26a significantly improved blood glucose tolerance and insulin sensitivity in diabetic mice. Moreover, miR-26a overexpression increased trabecular and cortical bone volume in diabetic mice by decreasing bone resorption and increasing bone formation, effects that were strictly dependent on the insulin receptor on osteoblasts. This demonstrates the importance of miR-26a and suggests that it may have a dual function to ameliorate diabetes development and improve bone quality aspects [41] (Fig. 1).

miRNA expression patterns were also assessed during fracture healing in diabetic rats. A transverse fracture was performed in the femur of 58 diabetic and 58 non-diabetic rats. In both groups, the post-fracture healing was assessed on day 5 and day 11. From the fracture healing area, they reported on day 5 14 miRNAs (miR-451-5p, miR-532-5p, miR-551b-3p, miR-339-3p, miR-181a-1-3p, miR-3065-3p, miR-222-3p, miR-133a-5p, miR-181b-5p, miR-3593-3p, miR-183-3p, miR-125b-1-3p, miR-6324, miR-151-3p) and on day 11 17 miRNAs (miR-379-3p, miR-376a-3p, miR-221-3p, miR-379-5p, miR-6216, miR-210-3p, miR-6324, miR-675-3p, miR-330-3p, miR-455-3p, miR-6317, miR-3557-3p, miR-455-5p, miR-207, miR-3075, miR-140-5p, miR-140-3p) differentially expressed between diabetic and non-diabetic rats. This investigation demonstrates that miRNA expression levels dynamically change over time. According to previously published papers, they further validated the deregulation of five miRNAs including miR-140-3p, miR-140-5p, miR-181a-1-3p, miR-210-3p, and miR-222-3p. However, further animal experiments are required to determine the causal role of those miRNAs in T1DM bone disease [42] (Fig. 1).

## The Role of miRNA in T2DM and Bone Pathology

T2DM is a disease with multiorgan complications. The last decade research has revealed that bone homeostasis is affected by crosstalk with the diabetic environment. It is now well-established that T2DM patients have increased risk of bone fractures, despite having increased BMD [6, 7, 8•]. This indicates that the diabetic bone quality is different and less resistant to fractures despite the normal to increased BMD. Mechanistically, T2DM is associated with a low bone turnover, which may contribute to the failure to renew micro-damage that naturally occurs during life, thereby, leading to the accumulation of structurally weak bone. Moreover, increased concentrations of advanced glycation end-products have been detected in diabetic bone, which may contribute to stiffer collagen structures and brittle bone. In particular, bone formation is profoundly suppressed under diabetic conditions. Among other, inhibition of Wnt signaling appears to contribute to reduced osteogenesis and increased adipogenesis. Also, increased oxidative stress has been shown to contribute to diabetic bone disease. As miRNAs control osteoblast and osteoclast differentiation and show great potential as biomarkers for diabetes and osteoporosis, they may be important mediators and/or predictors of diabetic bone disease.

To evaluate if the miRNA expression profile is distinct from individuals with fractures to individuals without fractures in patients with T2DM and postmenopausal osteoporosis, a miRNA-qPCR array was utilized. qPCR array analysis identified 48 differentially expressed miRNA between fractured and non-fractured individuals with a T2DM background and only 23 miRNAs were differently expressed in non-diabetic individuals with an osteoporotic background with and without fractures. miR-550a-5p and miR-382-3p were identified being the most promising circulating miRNA for diabetic bone disease, while miR-382-2p and miR-188-3p were discovered to be the most promising miRNA for osteoporosis. Furthermore, to investigate the effect of miR-550a-5p, miR-188-3p, and miR-382-3p on osteogenesis, adipogenesis, and cell proliferation, an in vitro functional study was performed in human adipocyte tissue–derived mesenchymal stem cells. The study revealed miR-382-3p significantly enhanced osteogenic differentiation whereas miR-550a-5p inhibited this process. miR-382-3p and miR-550a-5p impaired adipogenic differentiation whereas miR-188-3p did not show an effect on adipogenesis. All three miRNAs did not show any significant effect on cell proliferation (Fig. 1). The effects of miR-382-3p, miR-550a-5p, and miR-188-3p on osteoclastogenesis were not studied and require further investigation. Altogether, these results highlight miR-550a-5p and miR-382-3p as potential candidates for the indication of fragility status in the human T2DM cohort, while miR-188-3p potentially could be used as an indicator for osteoporosis-associated fractures. However, it should be noted that these miRNAs were measured in the serum only, and not at the tissue level [43•].

BMD and FRAX scores tend to underestimate fracture risk in patients with T2DM. A recent study investigated the combination of miRNA and the fracture risk assessment tool for the prediction of fracture risk. miRNAs were extracted from baseline serum from 168 postmenopausal women from the AGES-Reykjavik cohort and 10 panels of circulating miRNA known to be involved in diabetic bone disease and aging were validated with qPCR. Covariates included age, BMI, aBMD, clinical FRAX, and FRAX with aBMD. Three miRNAs were linked with the incidence of diabetic fragility fractures. miR-19b-1-5p was associated with lower incident diabetic fragility fracture, while postmenopausal women with high circulating levels of miR-203a and miR-31-5p had increased risk of fracture. Lastly, to improve fracture risk prediction, covariates and all three miRNAs were combined. This combination predicted fractures better than using the fracture assessment prediction aBMD alone, thus, suggesting that incorporating miRNA signatures could improve fracture risk prediction in patients with diabetes [44].

Recent studies have shown that T2DM compromises bone fracture healing. In this context, the role of miR-222, which is an important regulator of fracture healing, was investigated during mid-diaphyseal fracture healing in a rat model of T2DM. miR-222 was reciprocally altered during bone fracture healing. T2DM fractured rats displayed high levels of miR-222. miR-222 levels returned to normal levels after bone healing in non-diabetic fractured rats, demonstrating that T2DM rats had a longer bone healing period and that diabetes could inhibit bone healing. Furthermore, treating rat mesenchymal stem cells with miR-222 mimic suppressed osteogenesis, while it was increased after treatment with a miR-222 inhibitor. Thus, miR-222 could be a potential target for accelerating bone healing [45•], potentially also during diabetic conditions (Fig. 1).

Stearoyl-coenzyme A desaturase (SCD1) inhibits the development of diabetic bone disease by promoting osteogenesis. To assess if this regulation is achieved by specific miRNA, a SCD1-miRNA-mRNA network was established in bone marrow samples derived from postmenopausal women with diabetes aged 55–70 years old where 20 women with low-energy lower-limb fracture and 10 healthy premenopausal with high-energy lower-limb fracture were included. SCD1 expression was downregulated in bone marrow of patients with T2DM and low-energy fracture compared to the other group. Further, SCD1 regulates miR-203a and miR-1908, which control the expression of FOS and EXO1 and may be associated with diabetic fracture. Furthermore, miR-943, miR-550a-5p, miR-382-3p, and miR-376c-3p were also identified as regulators of not only FOS and EXO1, but also PLS1 and CDKN1A which could explain how T2DM exerts negative effects on bone [46]. Hence, SCD1 could be beneficial for the treatment of diabetic patients at high risk of fractures.

miR-205 negatively impacts osteogenic differentiation of bone marrow stromal cells by directly targeting Runx2. To investigate the expression level of miR-205 in a diabetic bone context, bone specimens from 60–80-year-old women with T2DM complicated with osteoporosis and a control group consisting of individuals with normal glycemic control and normal BMD were examined for miR-205 expression level by RT-PCR. miR-205 was highly expressed in women with T2DM and osteoporosis compared to women without T2DM and osteoporosis. In line with the human data, miR-205 expression level was also higher in mice with T2DM bone loss compared to controls. Finally, overexpression of miR-205 in bone marrow mesenchymal stem cells isolated from the diabetic animals inhibited osteogenesis and promoted adipogenic differentiation, while miR-205 knocked down promoted osteogenesis and inhibited adipogenic differentiation. This demonstrates miR-205 can regulate osteogenic/adipogenic differentiation potentially paving the way for a new therapeutic target treating patients with T2DM and osteoporosis [47] (Fig. 1).

The Wnt signaling pathway is an evolutionary conserved signal transduction pathway, which plays a vital role in regulating processes such as tissue proliferation and bone formation. Numerous studies have demonstrated that dysregulations in the Wnt signaling pathway represent a fundamental mechanism of various bone diseases. Also in the context of diabetic bone disease, several Wnt proteins and inhibitors are dysregulated. In vitro studies have identified multiple miRNA binding partners of key Wnt signaling components, LRP-6 (miR30e-5p), DKK1 (miR-152-3p, miR-335), and APC (miR-27a-3p, miR-142). Transcriptome analysis in a diabetic osteoporotic rat model revealed Wnt, AGE-RAGE, and PI3K-Akt to be highly enriched signaling pathways. Furthermore, qPCR revealed four main miRNAs (miR-124-3p, miR-184, miR-200a-3p, and miR-138-5p); both miR-124-3p and miR-184 were downregulated in rats with diabetes and osteoporosis compared to the control group. No significant changes were seen for miR-200a-3p and miR-138-5p. miR-124-3p had a profound expression profile and was chosen for further analysis. High glucose treatment in bone marrow stromal cells from diabetic and osteoporotic rats reduced expression of miR-124-3p and gene expression of Alp1 and Runx2 inhibiting osteogenesis (Fig. 1). Overexpression of miR-124-3p reversed the inhibitory effect. Hence, miR-124 might be a potential candidate to overcome suppressed osteogenesis under high glucose conditions [48].

miR-493-5p, another miRNA related to the Wnt/β-catenin pathway, plays a vital role in several cell biological functions. miR-493-5p’s downstream target is ZEB2, which activates Wnt/β-catenin and induces osteogenesis. How this interaction is regulated in diabetic osteoporosis is unknown. Human bone marrow stromal cells isolated from healthy volunteers were treated with low and high glucose. Low glucose treatment reduced the expression of miR-493-5p and was reversed when treated with high glucose, suggesting hyperglycemia affects miR-493-5p inhibiting Wnt/β-catenin. To support their findings, knockdown experiments were conducted in C57BL/6J diabetic osteoporotic mice and compared to the respective control. Knockdown of miR-493-5p alleviated diabetic osteoporosis in vivo and induced the expression of ZEB2, activating the Wnt pathway and prompting osteogenesis. Conclusively, hyperglycemia prevents osteogenesis by upregulating miR-493-5p thereby reducing ZEB2 and deactivating Wnt pathway (Fig. 1). miR-493-5p shows great promises as a potential therapeutic target and provides a novel strategy for diabetic osteoporosis [49].

In the perspective of diabetic osteoporosis, miR-29c-3p was measured in bone tissue from a rat model of diabetic osteoporosis. miR-29c-3p was significantly lower in the diabetic osteoporosis group compared to healthy rats. The protein expression of disheveled 2 (Dvl2), which is a downstream target involved in Wnt/β-catenin pathway, was significantly higher in the bones of diabetic rats. Using miR-29c-3pm mimics in diabetic rats, miR-29c-3p levels could be enhanced to control levels, while Dvl2 expression was reduced, suggesting that miR-29c-3p modulates Dvl2 expression. All these findings suggest that an overexpression of miR-29c-3p can exert a protective effect in diabetic osteoporosis and reduce bone loss by suppressing Dvl2. These findings provide a potential therapeutic target for diabetic osteoporosis [50].

## Perspectives and Challenges of miRNAs as Biomarkers in Diabetic Bone Disease

Taken together, miRNAs offer promising tools to treat diabetic bone disease. Several miRNAs were identified in recent years that mediate diabetic bone disease and thus could be therapeutically targeted. Moreover, miRNAs hold great potential as biomarkers as they are readily detectable and stable in biological fluids such as blood and urine, which are easily accessible. Even though circulating miRNAs are not per se organ-specific, many appear to correlate with specific organ functions, such as in the case of diabetic bone disease, glucose levels, bone turnover, or fracture occurrence. Thus, identifying such circulating miRNAs would have the benefit of preventing invasive procedures such as tissue biopsies for assessing organ function. Moreover, using well-characterized prospective cohorts, circulating miRNAs could be identified that can aid in the early diagnosis of diabetes before irreversible damage occurs. However, before miRNAs can be used as biomarkers, many challenges must be overcome. First, replicating studies in larger cohorts is needed to confirm previous findings. Although miRNAs are considered stable, independent studies often cannot identify the same miRNA profiles, which may be due to the use of different analytical and pre-analytical procedures including sample collection, miRNA extraction procedure, and storage conditions. Therefore, standardized operating procedures and references could be key to successfully identify and validate miRNAs across different laboratories. Moreover, most studies to date focus on miRNA identification under established diabetic conditions. However, it would important to consider the different stages of diabetes progression to cover all aspects of disease development and potentially identify stage-specific miRNA biomarkers. By defining the disease stage properly (as well as other parameters that define cohorts such as age, sex, and treatments), the results may also be more consistent.

Another way to use miRNAs as biomarkers for fracture prediction may be to use panels of miRNAs. Therein, an OsteomiR panel was used to evaluate the power to predict bone fractures in older people (not related to diabetes) consisting of 20 miRNA and 5 controls [51]. miRNA was extracted from serum of 17 individuals that had developed a bone fracture within 3 years and from serum of 16 individuals who had not experienced any bone fracture. Of the 20 miRNAs, 10 miRNAs were employed to calculate the OsteomiR score. A higher OsteomiR score was found in individuals who experienced fractures compared to control subjects with a predictive value of 66% and a sensitivity of 76%. The OsteomiR score was higher in osteopenic and osteoporotic subjects compared to subjects with a normal T score. Additionally, the OsteomiR score predicted more fracture events than the recommended “need-to-treat” threshold based on FRAX 10-year probability. A similar benefit of combining a miRNA panel with FRAX was identified in a study including patients with diabetes and fractures [44], underlining that such a combinatorial approach may be more suitable to predict fractures than FRAX alone.

Thus, even though studies indicate that miRNAs may be new tools to help identify patients at risk for fracture, it is mandatory to validate current results with small sample sizes in larger and more well-defined cohorts. Such cohorts may also allow for properly accounting for various confounders and organ interactions, especially at late stages of disease. Moreover, results from animal studies ought to be verified in human cohorts, as they only reflect specific stages of disease and often do not faithfully reflect all complications of diabetes (e.g., development of atherosclerosis). Nonetheless, miRNAs hold great potential as biomarkers for the prediction of fractures in patients with diabetes; thus, validation studies are highly encouraged.

## Conclusion

miRNAs play a critical role in the development of diabetic bone disease by controlling bone cell actions under high glucose conditions. However, more experimental studies are needed to define the causal relationships between miRNAs and diabetic bone disease before miRNAs may be considered as therapeutic targets. Further, perspective studies with larger sample sizes are required to assess the potential of miRNAs as biomarkers for predicting fracture risk in patients with diabetes.
